# A dominant role for the methyl-CpG-binding protein Mbd2 in controlling Th2 induction by dendritic cells

**DOI:** 10.1038/ncomms7920

**Published:** 2015-04-24

**Authors:** Peter C. Cook, Heather Owen, Aimée M. Deaton, Jessica G. Borger, Sheila L. Brown, Thomas Clouaire, Gareth-Rhys Jones, Lucy H. Jones, Rachel J. Lundie, Angela K. Marley, Vicky L. Morrison, Alexander T. Phythian-Adams, Elisabeth Wachter, Lauren M. Webb, Tara E. Sutherland, Graham D. Thomas, John R. Grainger, Jim Selfridge, Andrew N. J. McKenzie, Judith E. Allen, Susanna C. Fagerholm, Rick M. Maizels, Alasdair C. Ivens, Adrian Bird, Andrew S. MacDonald

**Affiliations:** 1 Manchester Collaborative Centre for Inflammation Research, University of Manchester, Manchester M3 9NT, UK; 2 Wellcome Trust Centre for Cell Biology, University of Edinburgh, Edinburgh EH9 3BF, UK; 3 Institute of Immunology and Infection Research, Centre for Immunity, Infection and Evolution, School of Biological Sciences, University of Edinburgh, Edinburgh EH9 3FL, UK; 4 Medical Research Institute, Ninewells Hospital and Medical School, University of Dundee, Dundee, DD1 9SY UK; 5 Medical Research Council Laboratory of Molecular Biology, Cambridge, CB2 0QH UK

## Abstract

Dendritic cells (DCs) direct CD4^+^ T-cell differentiation into diverse helper (Th) subsets that are required for protection against varied infections. However, the mechanisms used by DCs to promote Th2 responses, which are important both for immunity to helminth infection and in allergic disease, are currently poorly understood. We demonstrate a key role for the protein methyl-CpG-binding domain-2 (Mbd2), which links DNA methylation to repressive chromatin structure, in regulating expression of a range of genes that are associated with optimal DC activation and function. In the absence of Mbd2, DCs display reduced phenotypic activation and a markedly impaired capacity to initiate Th2 immunity against helminths or allergens. These data identify an epigenetic mechanism that is central to the activation of CD4^+^ T-cell responses by DCs, particularly in Th2 settings, and reveal methyl-CpG-binding proteins and the genes under their control as possible therapeutic targets for type-2 inflammation.

## Abstract (web summary)

How anti-helminth and allergic immune responses are initiated is poorly understood. Here the authors show that to trigger these responses, dendritic cells specifically require methyl-CpG-binding domain-2, a protein promoting repressed chromatin state.

## Graphical abstract

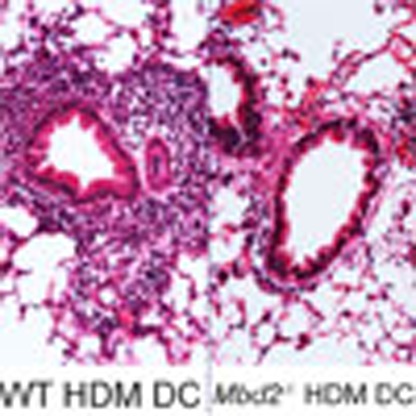

Dendritic cells (DCs) are specialized innate immune cells with an unparalleled ability to respond to inflammation and pathogens and initiate adaptive T-cell immunity^1^. In this antigen-presenting cell (APC) role, DCs are centrally involved in directing the character of the developing CD4^+^ T-cell response, influencing the range and dominance of the cytokines they produce^2^.

Type-2 immunity is a defining feature of allergic responses and parasitic helminth infection^3^,^4^. Although T-helper (Th)2 cytokines can mediate protection and wound healing in the context of extracellular pathogens such as helminths, excessive Th2 inflammation can cause substantial damage to the host in either helminth infection or allergic disorders^5^. It is clear that DCs are required for Th2 priming in both of these settings^6^,^7^,^8^. However, the specific molecular mechanism(s) that they employ to induce Th2 responses are poorly understood and much debated^3^,^4^,^9^.

Exposure of DCs to bacterial, viral or protozoal antigens triggers their dynamic activation and the release of pro-inflammatory cytokines that are vital for Th1/Th17 T-cell polarization^1^. In contrast, a hallmark of Th2-inducing DCs is a low-level or muted activation, distinct from that of Th1/17 DCs^2^,^9^. In particular, helminths generally fail to provoke DC pro-inflammatory cytokine release and induce minimal changes in DC messenger RNA (mRNA) expression profiles^9^. Perhaps, because of this, one theory that has been proposed is that Th2 induction may represent a ‘default’ pathway that occurs when DCs fail to be markedly activated^10^. However, a range of molecules have been associated with the ability of DCs to generate optimal Th2 immunity, including CD40 (ref. 11), CD80/86 (ref. 12), OX40L^13^,^14^, CCL17 (ref. 15), RELMα^16^, ERK, c-Fos^17^ and NF-κB^18^. In addition, the transcription factors Irf4 (refs 19, 20) and STAT5a/JAK2 (ref. 21) have recently been suggested to be important for successful Th2 induction by DCs. Collectively, this highlights that Th2 priming by DCs responding to allergens or helminths is a complex process, and that our current understanding of the specific and dominant regulatory mechanisms involved is incomplete.

In recent years, it has become clear that ‘epigenetic’ mechanisms, which alter gene expression without changing underlying DNA sequence, play an important role in regulating multiple aspects of T-cell differentiation and function^22^,^23^. Although much less is known about epigenetic control of innate cells, it has recently been shown that histone methylation can regulate fibroblast and DC antiviral responses^24^, as well as myeloid cell differentiation and activation^25^.

Methyl-CpG-binding proteins are required for normal gene regulation during development^26^,^27^. The methyl-CpG-binding domain protein, Mbd2, links DNA methylation to transcriptional silencing via the nucleosome remodelling and histone deacetylase (NuRD) complex^28^. Although Mbd2 is widely expressed in immune cells^29^ and has previously been implicated in control of T-cell differentiation^30^,^31^,^32^,^33^, no role has yet been identified for it in innate immune cells such as DCs.

We have assessed whether epigenetic control of gene expression is important for DC activation and function, and in the promotion of Th2 responses. Our results reveal that Mbd2 regulates DC expression of a suite of immunologically relevant genes and plays a dominant role in regulating the ability of DCs to prime type-2 responses *in vitro* and *in vivo*, against either helminths or allergens. This requirement for Mbd2 provides further evidence that Th2 priming by DCs is more than a passive ‘default’ process and highlights methyl-binding domain proteins, and the genes under their control, as novel therapeutic targets for Th2 inflammation.

## Results

### Mbd2 regulates key DC pathways associated with APC function

**Figure 1:**
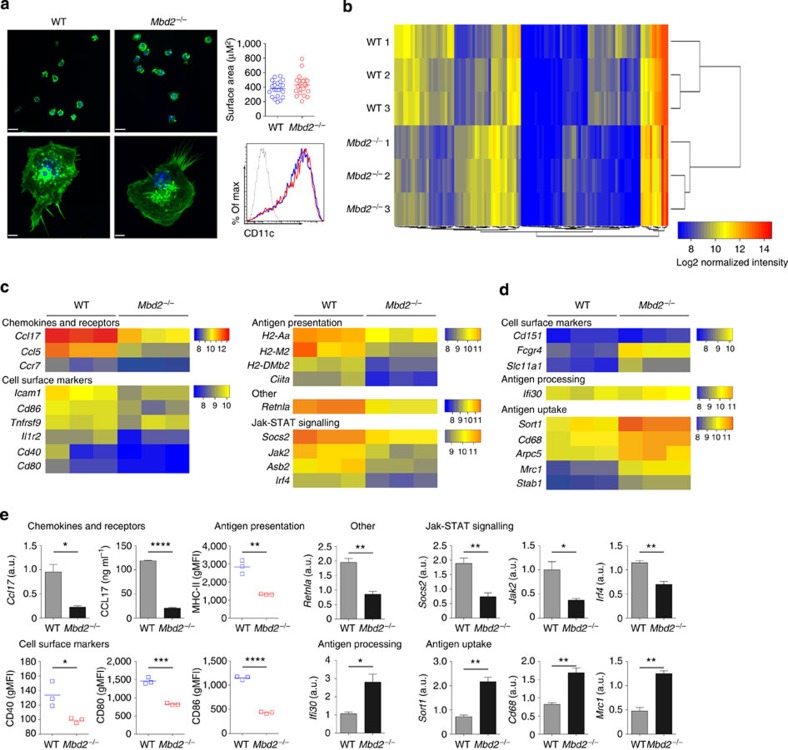
Mbd2 regulates expression of several key DC pathways associated with APC function. (**a**) WT or *Mbd2*^*−/−*^ BMDCs cultured on multichamber glass slides were stained with phalloidin (green) and 4,6-diamidino-2-phenylindole (blue) and surface area analysed by confocal microscopy. Photomicrographs are representative images from five fields in one experiment of three (scale bars, 38 μM (top panel) and 7 μM (bottom panel)). CD11c staining on WT (blue) and *Mbd2*^*−/−*^ (red) BMDCs (one of six experiments) was assessed by flow cytometry. (**b**) Heat map showing the mRNA signature of WT versus *Mbd2*^*−/−*^ BMDCs (119 genes, log2 normalized intensity, twofold change-filtered, *P*<0.05 (moderated *t*-test), three biological replicates per genotype). (**c**) Heat map showing 18 downregulated *Mbd2*^*−/−*^ versus WT BMDCs genes selected based on putative function following network analysis. (**d**) Heat map showing nine upregulated *Mbd2*^*−/−*^ versus WT BMDCs genes selected based on putative function following network analysis. (**e**) To validate microarray data, mRNA expression of genes of interest were assessed by qPCR (normalized against *Hprt*, a.u.), surface protein expression measured by flow cytometry and secreted protein by ELISA, comparing WT and *Mbd2*^*−/−*^ BMDCs. Results are mean+s.e.m. (three replicate wells, one of at least six experiments). *P<0.05, **P<0.01, ***P<0.001, ****P<0.0001 (Student’s *t*-test). a.u., arbitrary units; gMFI, geometric mean fluorescence intensity.

To determine the importance of Mbd2 in DC development and function, we generated bone marrow-derived DCs (BMDCs) from wild-type (WT) and *Mbd2*^*−/−*^ mice and compared their mRNA expression profiles (Fig. 1). Although they developed similarly to WT *in vitro* and *in vivo* (Fig. 1a; Supplementary Fig. 1), *Mbd2*^*−/−*^ BMDCs displayed strikingly altered mRNA expression, with 70 genes significantly downregulated (>twofold, *P*<0.05) and 49 genes significantly upregulated (>twofold, *P*<0.05), compared with WT (Fig. 1b; Supplementary Data 1). KEGG (Kyoto Encyclopedia of Genes and Genomes) pathway and GO (Gene Ontology) term analysis of the *Mbd2*^*−/−*^ BMDC mRNA signature identified transcripts associated with several pathways crucial for DC function (Fig. 1c,d; Supplementary Data 2). A range of these expression changes was verified by the analysis of mRNA (quantitative reverse transcription PCR (qRT–PCR)) and protein levels (flow cytometry and enzyme-linked immunosorbent assay (ELISA); Fig. 1e). Further, many of the gene expression and phenotypic differences evident in immature *Mbd2*^*−/−*^ BMDCs (Fig. 1) were also apparent following their exposure to strong Th2 or Th1/17 antigens (soluble egg antigen (SEA) from the parasitic helminth *Schistosoma mansoni*, or heat-killed *Salmonella typhimurium* (St) (Supplementary Fig. 2). Transcripts that were downregulated in *Mbd2*^*−/−*^ BMDCs encompassed several important immunological processes, including antigen presentation (*H2-Aa*, *Ciita*) and co-stimulation (*Tnfrsf9* (4-1BB), *Cd40*, *Cd80* and *Cd86*). Since these molecules are directly required for T-cell activation by APCs^2^, this suggested that *Mbd2*^*−/−*^ DCs may be less able to initiate CD4^+^ T-cell responses. Pathway analysis of mRNA transcripts upregulated in *Mbd2*^*−/−*^ BMDCs identified genes such as *Mrc1* (mannose receptor), *Stab1* (stabilin receptor), *Cd68*, *Slc11a1* and *Ifi30*, all of which are primarily linked with antigen uptake and processing pathways^34^,^35^,^36^,^37^,^38^,^39^. This demonstrates that Mbd2 is crucial for governing the optimal expression of genes that are important for a variety of DC functions, many of them related to APC ability.

**Figure 2:**
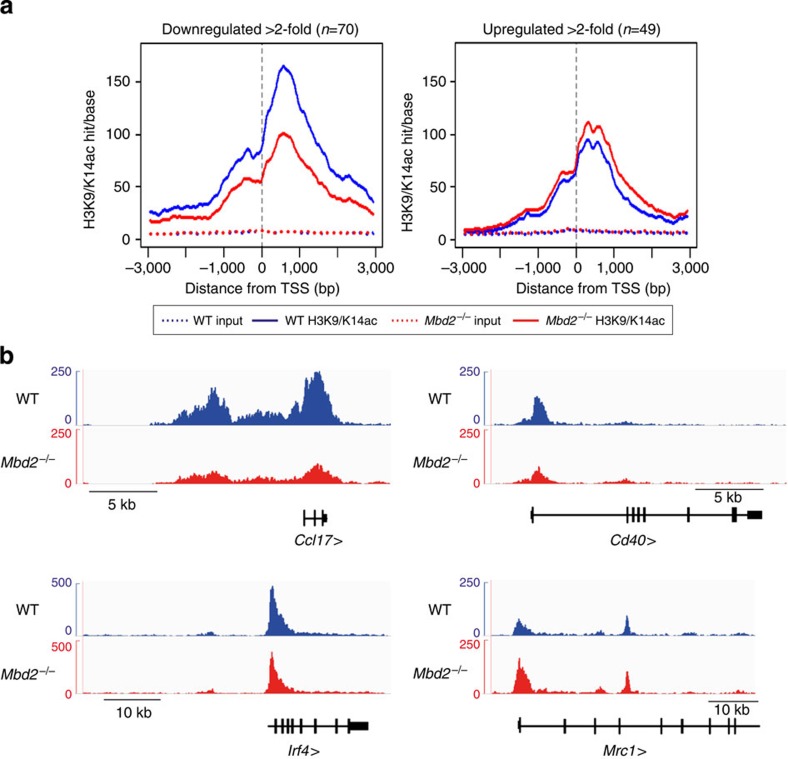
H3K9/K14 acetylation of genes controlled by Mbd2. (**a**) ChIP sequencing composite profile showing H3K9/K14ac or input DNA signal (as read coverage) for WT (blue) and *Mbd2*^*−/−*^ (red) BMDCs for a selection of genes with significantly altered mRNA profiles. Signal is displayed from −3 kb to +3 kb surrounding each annotated transcription start site (TSS). (**b**) Genome screenshots representing H3K9/K14ac signal in WT (blue) and *Mbd2*^*−/−*^ (red) BMDCs at selected gene loci (*Ccl17*, *Cd40, Irf4* and *Mrc1*). RefSeq genes are in black.

A recent study using biotin-tagged Mbd2 and mouse embryonic stem cells confirmed that the primary determinant of Mbd2 binding is methylated-CpG dinucleotides^40^. Thus, most Mbd2 is not targeted to specific genomic locations, but globally tracks the density of DNA methylation, which occurs on average once per 150 bp (ref. 40). Mbd2 acts as a reader of the methyl-CpG signature, then recruits the NuRD co-repressor complex, to reinforce transcriptional silencing through the nucleosome remodelling and histone deacetylase activity of NuRD^26^,^40^. As available antibodies, including those generated in-house, did not permit chromatin immunoprecipitation (ChIP) sequencing analysis of Mbd2 in DCs (data not shown), we instead used an antibody against H3K9/K14ac, an epigenetic marker of active gene transcription^22^,^28^, to assess the levels of histone acetylation near transcriptional start sites (TSSs) of significantly down- or upregulated genes from *Mbd2*^*−/−*^ BMDCs in comparison with WT. Genes for which mRNA expression was decreased by twofold or greater in *Mbd2*^*−/−*^ BMDCs showed reduced H3K9/K14 acetylation at their TSS (Fig. 2a; Supplementary Fig. 3; Supplementary Data 3), and this was particularly evident for *Ccl17*, *Cd40* and *Irf4* (Fig. 2b; Supplementary Fig. 3b). In contrast, upregulated genes showed unaltered H3K9/K14 acetylation in *Mbd2*^*−/−*^ BMDCs (Fig. 2a; Supplementary Fig. 3; Supplementary Data 4). This indicates that Mbd2 controls DC expression of a wide range of immunologically relevant genes, and that this process may involve modulation of H3K9/K14 acetylation.

### Mbd2 expression is required for optimal DC function

We next addressed whether these alterations in H3K9/K14 acetylation, which accompanied decreased expression of genes associated with antigen presentation and co-stimulation of T cells and increased expression of genes associated with antigen uptake (Figs 1 and 2; Supplementary Fig. 3), translated into altered DC function.

**Figure 3:**
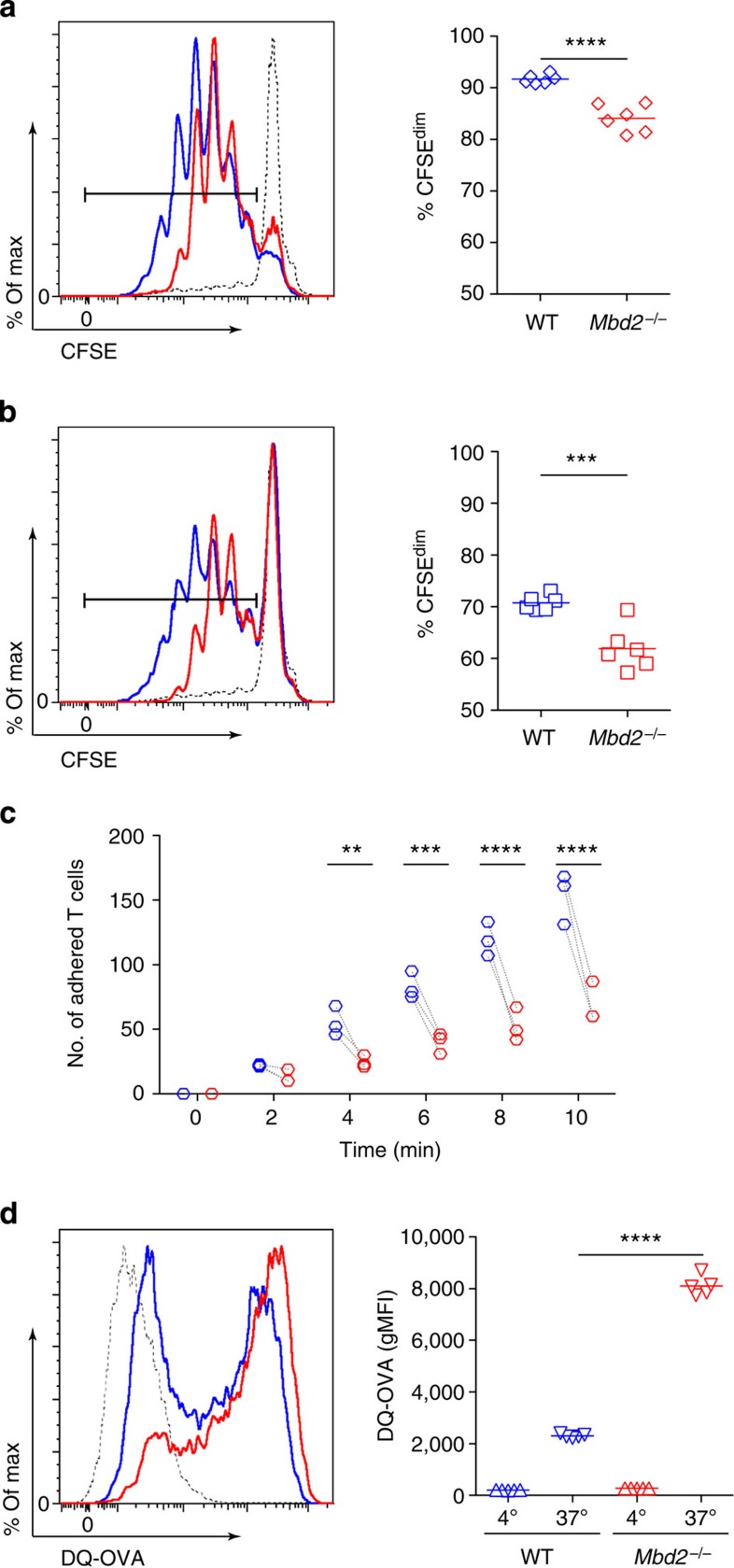
Mbd2 regulates DC antigen uptake and interaction with CD4^+^ T cells. (**a**,**b**) WT (blue) or *Mbd2*^*−/−*^ (red) BMDCs were cultured for 4 days with CFSE-labelled OT-II T cells in the presence of OVA peptide (**a**) or protein (**b**). T-cell proliferation was assessed by flow cytometric analysis of CFSE dilution (six wells per group, one of five experiments). (**c**) Adhesion of WT CD4^+^ T cells to WT or *Mbd2*^*−/−*^ BMDCs under shear flow conditions (*n*=mean of T-cell adhesion combined from three separate experiments). (**d**) WT (blue) or *Mbd2*^*−/−*^ (red) BMDCs were incubated with DQ-OVA at 37 or 4 °C, and uptake assessed by flow cytometry (five wells per group, one of more than five experiments). **P<0.01, ***P<0.001, ****P<0.0001 (Student’s *t*-test (**a**,**b**) or ANOVA (**c**,**d**). gMFI, geometric mean fluorescence intensity.

To assess DC ability to stimulate T-cell proliferation *in vitro*, BMDCs were pulsed with ovalbumin (OVA) peptide, which binds directly to major histocompatibility complex-II (MHC-II), or OVA protein, which requires processing for presentation of OVA peptides on MHC-II, then cultured with OVA-specific OT-II CD4^+^ T cells. Analysis of OT-II proliferation showed that *Mbd2*^*−/−*^ BMDCs were significantly less able to stimulate T-cell division compared with WT BMDCs when pulsed with OVA peptide (Fig. 3a) or protein (Fig. 3b).

*Mbd2*^*−/−*^ BMDCs also displayed reduced expression of *Icam-1* at both the transcript and the protein level (Fig. 1c). ICAM-1 (intercellular adhesion molecule 1) has been shown to be important for controlling the duration of DC–T-cell interactions^41^, and *Mbd2*^*−/−*^ BMDCs bound a significantly reduced number of activated CD4^+^ T cells compared with WT BMDCs under conditions of shear flow *in vitro* (Fig. 3c).

In addition to decreased expression of molecules associated with T-cell interaction and stimulation, *Mbd2*^*−/−*^ BMDCs showed increased expression of a range of mRNA transcripts related to antigen uptake (Fig. 1). To determine the functional impact of increased expression of these genes, DCs were cultured with DQ-OVA, which is internalized by macropinocytosis via *Mrc1* (ref. 42). In keeping with their increased *Mrc1* mRNA expression (Fig. 1), *Mbd2*^*−/−*^ BMDCs displayed significantly increased capacity to take up DQ-OVA compared with WT BMDCs (Fig. 3d).

Together, these data reveal that Mbd2 is centrally involved in the ability of DCs to take up, process and present antigen and activate CD4^+^ T cells, highlighting the importance of epigenetic regulation, via methyl-binding domain proteins, in controlling optimal DC APC function.

### CD11c^+^ cell Mbd2 is vital for optimal Th2 responses

**Figure 4:**
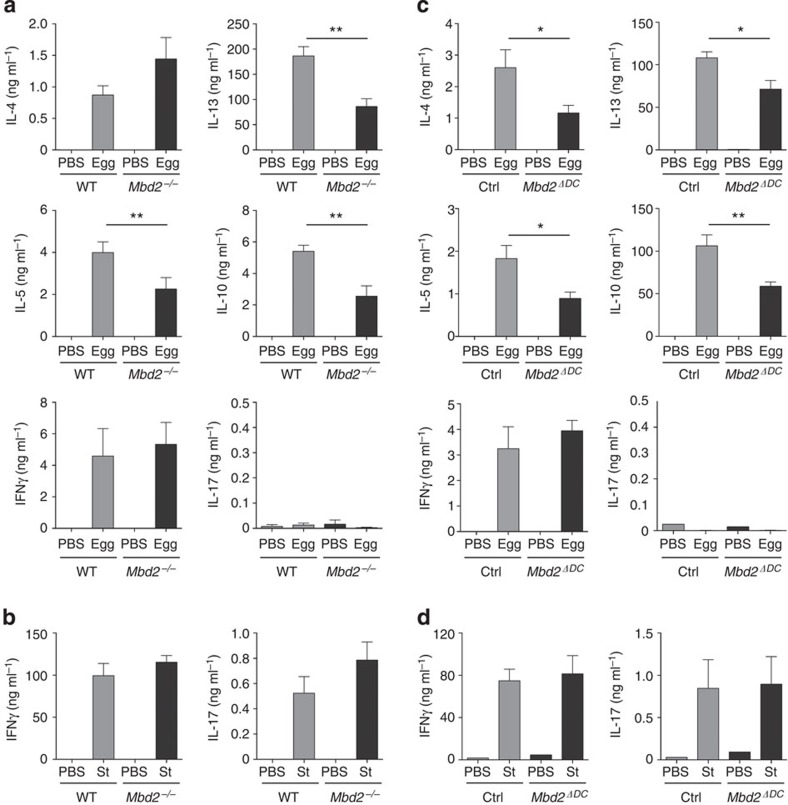
Mbd2 is vital for optimal Th2 but not for Th1 induction *in vivo*. (**a**,**b**) WT (grey) or *Mbd2*^*−/−*^ (black) mice were injected s.c. with PBS, *S. mansoni* eggs (**a**) or heat-killed *S. typhimurium* (St) (**b**). Seven days later the draining pLNs were harvested, cells restimulated for 72 h with SEA or St and cytokine secretion assessed by ELISA (five mice per group, one of two to four experiments). (**c**,**d**) *Mbd2*^Δ*DC*^ (black) mice or littermate controls (grey) were injected s.c. with PBS, *S. mansoni* eggs (**c**) or heat-killed *S. typhimurium* (St) (**d**). Seven days later the draining pLNs were harvested, cells restimulated for 72 h with SEA or St and cytokine secretion assessed by ELISA (five to six mice per group, one of two to three experiments). Bar graphs show mean+s.e.m. *P<0.05, **P<0.01 (Student’s *t*-test). pLNs, popliteal lymph nodes; s.c., subcutaneously.

Having identified that Mbd2 plays a key role in regulating the multiple aspects of basic DC function, we next wanted to specifically assess its importance during CD4^+^ T-cell polarization. To first determine the general influence of Mbd2 over this process, we injected *Mbd2*^*−/−*^ mice with eggs from *S. mansoni* (a potent Th2 stimulus)^8^ or heat-killed St (for promotion of Th1/17 polarization)^43^ and measured responses in the draining lymph nodes (LNs) 7 days later. As in many complex Th2 settings, along with Th2 cytokines, *S. mansoni* eggs induce low-level interferon-γ (IFNγ), and minimal interleukin (IL)-17, in C57BL/6 mice^44^,^45^. While antigen-specific IL-4 was similar following *S. mansoni* egg injection of *Mbd2*^*−/−*^ mice, other Th2 cytokines were significantly decreased (Fig. 4a). Further, in contrast to Th2 cytokines, *Mbd2*^*−/−*^ mice showed intact IFNγ following injection with *S. mansoni* eggs (Fig. 4a) or IFNγ/IL-17 in response to St (Fig. 4b). This demonstrated that global deficiency of Mbd2 results in defective Th2 rather than Th1/17 polarization, but did not distinguish whether this is attributable to a direct or indirect effect of Mbd2 on T cells or DCs.

Several of the genes that we identified as being dysregulated in *Mbd2*^*−/−*^ BMDCs (Fig. 1) have previously been linked to Th2 priming (for example, *Cd40* (ref. 11), *Cd80/Cd86* (ref. 12), *Retnla*^16^, *Jak2* (ref. 21) and *Ccl17* (ref. 15)), suggesting that Mbd2 may play a key role in governing the ability of DCs to prime Th2 responses. To directly address this possibility, mice with *lox*P sites flanking the first exon of *Mbd2* were generated and bred with mice that express Cre recombinase in DCs (*CD11c-Cre*^*+*^)^46^ to delete Mbd2 in CD11c^+^ DCs (*Mbd2*^ΔDC^; Supplementary Fig. 4). *Mbd2* transcripts in sorted splenic CD11c^hi^ cells from *Mbd2*^ΔDC^ mice were reduced compared with *CD11c-Cre*^*−*^ littermate controls (Supplementary Fig. 4g), while splenic DC development was equivalent (Supplementary Fig. 4h). *Mbd2*^ΔDC^ mice displayed significantly impaired Th2 cytokine production in response to *S. mansoni* egg challenge (Fig. 4c), but unaltered IFNγ priming following egg injection (Fig. 4c) or IFNγ/IL-17 in response to St (Fig. 4d). As expected, *S. mansoni* eggs induced IL-17 at negligible levels in WT, *Mbd2*^*−/−*^ or *Mbd2*^ΔDC^ mice (Fig 4b,d). Thus, these data reveal that expression of Mbd2 by DCs is vital for promotion of optimal Th2 immunity, but is not fundamentally required for Th1/17 induction, *in vivo*.

**Figure 5:**
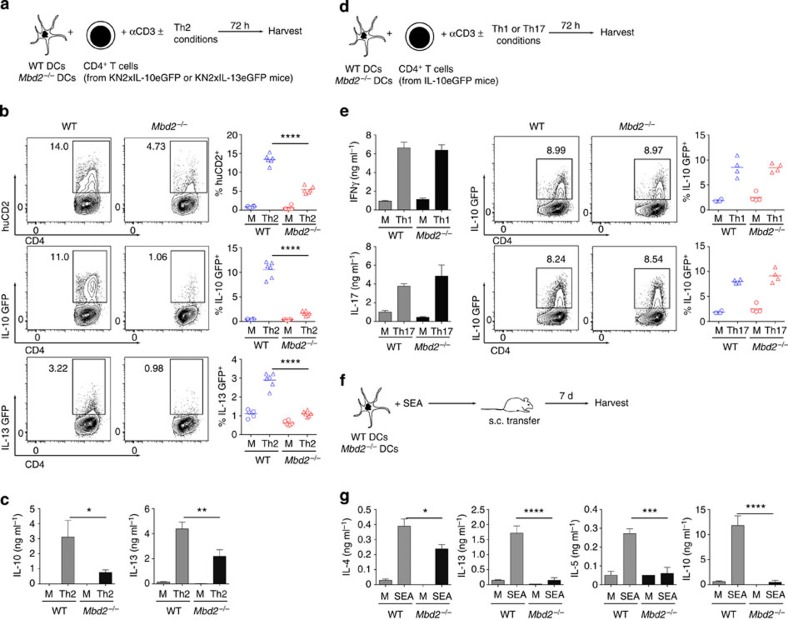
DC expression of Mbd2 is vital for optimal Th2 induction and development. (**a**,**b**) CD4^+^GFP^*−*^ T cells from KN2xIL-10eGFP or KN2xIL-13eGFP mice were cultured for 3 days with WT (blue) or *Mbd2*^*−/−*^ (red) BMDCs, anti-CD3 mAb±IL-4 (Th2 conditions) and assessed for IL-4 (huCD2) protein production, and IL-10 and IL-13 mRNA expression by flow cytometry (five to six replicate culture wells, one of six experiments). (**c**) CD4^+^GFP^*−*^ T cells from KN2xIL-13eGFP mice were cultured for 3 days with WT (grey) or *Mbd2*^*−/−*^ (black) BMDCs, anti-CD3 mAb±IL-4 (Th2 conditions) and supernatants assessed for IL-10 and IL-13 protein secretion by ELISA. (**d**,**e**) CD4^+^GFP^*−*^ T cells from IL-10eGFP mice were cultured for 3 days with WT (grey) or *Mbd2*^*−/−*^ (black) DCs, anti-CD3 mAb±IL-12 (Th1 conditions) or IL-6/TGF-β/IL-23 (Th17 conditions) and supernatants assessed for IFNγ and IL-17 protein secretion by ELISA or IL-10 mRNA expression was determined by flow cytometry (four replicate wells per group, one of three experiments). (**f**,**g**) WT (grey) or *Mbd2*^*−/−*^ (black) BMDCs were cultured overnight in medium alone (M) or with SEA and injected s.c. into WT mice. Seven days later the pLNs were harvested, cells restimulated for 72 h with SEA and cytokine secretion assessed by ELISA (four to five mice per group, one of three experiments). Bar graphs show mean+s.e.m. *P<0.05, **P<0.01, ***P<0.001, ****P<0.0001 (ANOVA). mAb, monoclonal antibody; s.c., subcutaneously; TGF-β, transforming growth factor-β.

Given the complexity of schistosome egg challenge *in vivo,* we next focused on the influence of Mbd2 on the ability of DCs to direct CD4^+^ T-cell polarization *in vitro*. WT BMDCs co-cultured with CD4^+^ T cells from KN2xIL-10eGFP (enhanced green fluorescent protein) or KN2xIL-13eGFP reporter mice^16^,^47^,^48^ (Fig. 5a) promoted eGFP (IL-10 or IL-13) or huCD2 (IL-4) expression (Fig. 5b), and secretion of IL-10 and IL-13 (Fig. 5c) in Th2 polarizing conditions. In comparison, *Mbd2*^*−/−*^ DCs displayed a marked impairment in Th2 inductive ability (Fig. 5b,c), while WT or *Mbd2*^*−/−*^ BMDCs induced similar IFNγ, IL-17 and IL-10 in Th1/Th17 settings (Fig. 5d,e), *in vitro*. In addition, *Mbd2*^*−/−*^ DCs pulsed with SEA from *S. mansoni* displayed a strikingly reduced ability to prime antigen-specific Th2 cytokines *in vivo* following adoptive transfer into naive WT recipients (Fig. 5f,g). Along with the data presented in Fig. 4, these results strongly suggest that DC expression of Mbd2 is particularly important for optimal Th2 response induction both *in vitro* and *in vivo*.

### DC Mbd2 is necessary for initiation of lung allergic inflammation

**Figure 6:**
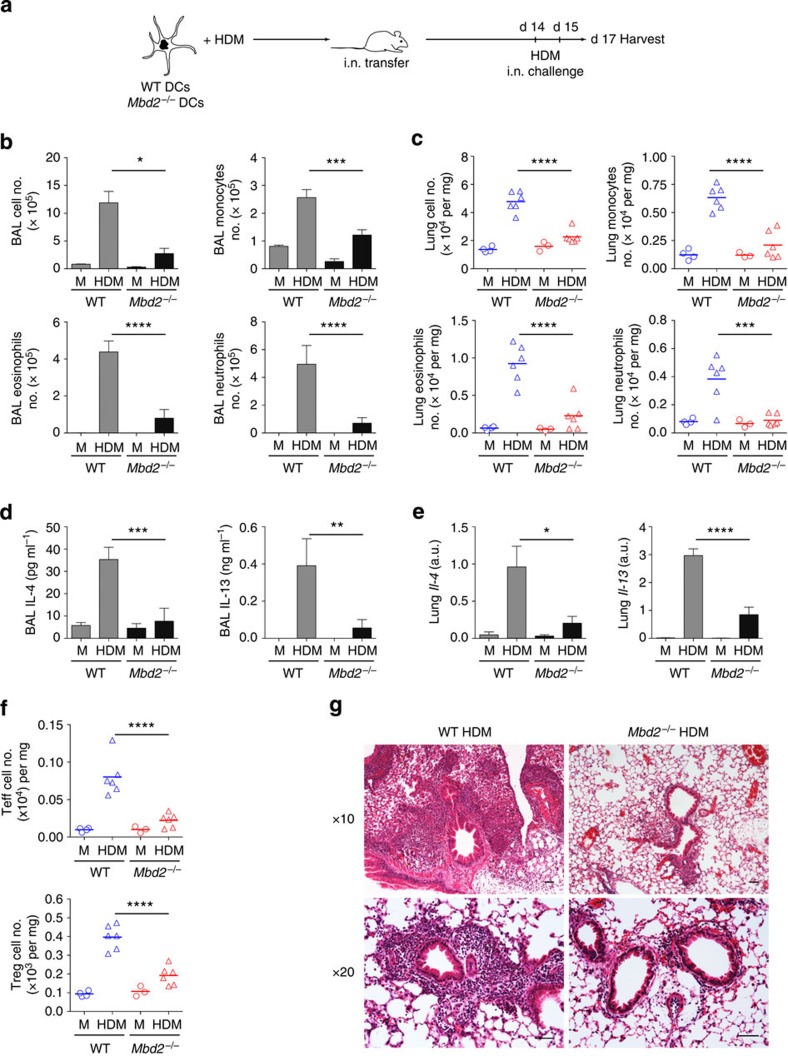
DC expression of Mbd2 is required for induction of pulmonary allergic inflammation. (**a**) WT mice were sensitized by intranasal administration of WT (blue or grey in **b**–**f**) or *Mbd2*^*−/−*^ (red or black in **b**–**f**) BMDCs that had been cultured overnight in medium alone (M) or with HDM, then intranasally challenged with 5 μg HDM on days 14 and 15 post BMDC transfer and tissues harvested on day 17. (**b**) BAL fluid cells were counted, cytospin preparations stained and 200 cells per slide counted for specific cell types. (**c**) Lung tissue cells were isolated and eosinophilia, neutrophilia and monocyte infiltration assessed by flow cytometry. (**d**) Cytokines in BAL fluid were measured by ELISA. (**e**) Lung tissue mRNA expression was assessed by qPCR (normalized against *Hprt*, a.u.). (**f**) Proportions of TCRβ^+^CD4^+^Foxp3^+^ Treg cells (Treg) and activated effector TCRβ^+^CD4^+^Foxp3^*−*^CD44^+^CD69^+^ T cells (Teff) in lung tissues were assessed by flow cytometry. (**g**) Representative lung sections from recipients of WT or *Mbd2*^*−/−*^ HDM-BMDCs, stained with hematoxylin and eosin (scale bar, 50 μM). Bar graphs show mean+s.e.m. (three to six mice per group, one of six experiments). *P<0.05, **P<0.01, ***P<0.001, ****P<0.0001 (ANOVA). a.u., arbitrary units.

House dust mites (HDM) are among the most common allergens in humans and potent inducers of bronchial inflammation in mice, with a strong Th2 involvement^49^,^50^. Sensitization by intranasal transfer of BMDCs that have been exposed to HDM (HDM-DCs), followed by intranasal challenge with HDM antigen, is a non-invasive system to induce murine allergic lung pathology (Fig. 6a)^51^,^52^. Using this approach, we next assessed whether Mbd2 is required for DCs to initiate Th2 allergic airway inflammation. WT HDM-BMDCs capably sensitized recipients to generate a robust inflammatory response on HDM challenge, with concomitant recruitment of mononuclear cells, neutrophils and eosinophils evident in bronchoalveolar lavage (BAL) fluid and lung tissue (Fig. 6b,c; Supplementary Fig. 5a). In contrast, recipients that had been sensitized with *Mbd2*^*−/−*^ HDM-BMDCs had significantly reduced numbers of these cells in BAL fluid and lung, and a significantly reduced Th2 cytokine response in BAL fluid and lung tissue (Fig. 6d,e). Impaired immune priming was also evident in reduced numbers of both effector and regulatory lung CD4^+^ T cells in mice sensitized with *Mbd2*^*−/−*^ HDM-BMDCs (Fig. 6f; Supplementary Fig. 5b). Finally, histology showed much less marked lung inflammation in mice sensitized with *Mbd2*^*−/−*^ HDM-BMDCs versus WT HDM-BMDCs (Fig. 6g). Thus, DC expression of Mbd2 is essential for initiation of a strong allergic pulmonary response against HDM *in vivo*.

## Discussion

Despite Th2 immunity mediating pathogenic allergic disorders and controlling protection to large extracellular pathogens, the central mechanisms that DCs employ to initiate Th2 polarization are poorly understood. We now provide a clear demonstration that epigenetic mechanisms play a dominant role in DC induction of type-2 immunity against both helminth antigen and allergens, regulating expression of key target genes. Furthermore, DC expression of Mbd2 appears to be less important in Th1/17 settings, as *Mbd2*^*−/−*^ BMDCs retained the ability to initiate Th1 and Th17 responses both *in vitro* and *in viv*o, even though they displayed a basally impaired capacity to stimulate proliferation of, and adhere to, CD4^+^ T cells *in vitro*.

Mbd2 is thought of as a reader of the mCpG signature that recruits or associates with the NuRD co-repressor complex, which in turn reinforces transcriptional silencing^26^,^40^. Thus, a surprising result from our work was that *Mbd2*^*−/−*^ BMDCs in fact displayed a larger number of downregulated, rather than upregulated, mRNA transcripts (Fig. 1). This suggests that Mbd2 is likely to function in a more dynamic manner than simply through direct transcriptional silencing of genes that impede Th2 promotion. Since many transcripts were significantly downregulated in *Mbd2*^*−/−*^ BMDCs (Fig. 1), some of which had an accompanying reduction in H3K9/K14 acetylation levels (Fig. 2; Supplementary Fig. 2), it is possible that Mbd2 may act in a more indirect fashion, regulating expression of transcriptional repressors that, when released from control in *Mbd2*^*−/−*^ DCs, reduce transcription of their target genes. However, this was not obviously the case, with the genes identified by pathway analysis as being significantly upregulated in *Mbd2*^*−/−*^ BMDCs being primarily associated with increased antigen uptake and processing, rather than inhibition of transcription (Fig. 1). Another alternative is that Mbd2 could enhance, as well as repress, gene expression in DCs. This dual function for Mbd2 would be in line with a recent study, which showed that genes bound by Mbd2 alone are transcriptionally repressed, while genes that are bound by both Mbd2 and Mbd3 (another methyl-CpG-binding domain family member) require Mbd2 for activation^53^. In this case, loss of Mbd2 alone could result in downregulated expression of that subset of target genes. These models are not mutually exclusive, and further work is required to identify which pathway dominates in Mbd2 regulation of DC function. Irrespective of this, our study provides strong evidence that DC-mediated induction of type-2 immunity is not a passive default process, instead requiring active regulation of gene expression.

Although displaying many similarities, the immunological events that lead to allergic responses are very different to helminth infections^3^,^5^, yet we have identified that both types of immune challenge require DC expression of Mbd2 for initiation of an optimal Th2 response. *Mbd2*^*−/−*^ BMDCs were less able to bind and activate CD4^+^ T cells *in vitro* (Fig. 3), likely due to reduced expression of Icam-1, MHC-II and co-stimulatory molecules (*Cd40*, *Cd80*/*Cd86* and *4-1BB*) (Fig. 1). While MHC-II, CD40 and CD80/86 have been previously shown to be required for DC-mediated Th2 induction^11^,^12^,^54^, our data now suggest new roles for ICAM-1 and 4-1BB in this process. Supportive of this possibility, ICAM-1-deficient animals display reduced Th2 cell numbers in a murine model of systemic sclerosis^55^, while antibody engagement of 4-1BB on T cells inhibits allergic responses^56^. In addition, *Mbd2*^*−/−*^ BMDCs showed reduced mRNA expression for the chemokines CCL5 and CCL17 (Fig. 1), chemoattractants involved during lung inflammation^52^. In particular, CD11b^+^ DC secretion of CCL17 is thought to be an important contributor to pulmonary allergic responses, required for recruitment of Th2 cells^15^. Our data highlight that Mbd2 regulates BMDC CCL17 expression and secretion (Fig. 1), supporting the importance of this chemokine for optimal Th2 immunity against either allergens or helminth antigens.

Analysis of *Mbd2*^*−/−*^ BMDCs also identified reduced mRNA expression of a range of genes involved in core signalling pathways, including *Jak2*, *Irf4* and *Socs2*. Both Jak2 and Irf4 have recently been implicated in DC Th2 function: Jak2 was shown to be important for STAT5a signalling in DCs and upregulating expression of co-stimulatory molecules, chemokines and their ability to induce Th2 responses following exposure to TSLP (thymic stromal lymphopoietin)^21^, while Irf4 may be a general requirement for efficient CD4^+^ T-cell priming^57^, with its conditional deletion in CD11c expressing cells reducing type-2 immunity to helminth infection^19^ and HDM elicited allergic responses^20^. Our study further supports the hypothesis that DCs require Jak2 and Irf4 to prime type-2 immunity, but provides the novel insight that expression of these signalling proteins is under epigenetic control by Mbd2. Furthermore, our data indicate that Socs2, which has previously been shown to influence DC responses to lipopolysaccharide by limiting STAT3 phosphorylation after TLR (Toll-like receptor) ligation^58^, may also play a key role, downstream of Mbd2, in modulating DC ability to prime Th2 cells.

In summary, we propose that Mbd2 controls low-level expression of a network of core molecules that together enable DC promotion of optimal Th2 immunity. Whether Mbd2/NuRD regulates expression of downstream co-stimulatory molecules and chemokines directly, or indirectly via signalling molecules such as Jak2, Irf4 and Socs2, remains a question requiring further study. However, our work goes beyond profiling to emphasize how epigenetic mechanisms can be crucial for determining not only activation status, but also function of innate immune cells. It also identifies in Mbd2 a single protein that is central for Th2 induction by DCs, providing a platform for future development of interventions targeting DCs, Mbd2 or associated proteins, to enable therapeutic manipulation of allergic inflammation and anti-helminth host defence.

## Methods

### Animals

*Mbd2*^*−/−*^ (ref. 59), KN2xIL-10eGFP^16^,^47^, KN2xIL-13eGFP^47^,^48^, IL-10eGFP^60^ and OT-IIxLy5.1 (ref. 16) mice all on a C57BL/6 background were maintained in the School of Biological Sciences, University of Edinburgh, or the Faculty of Life Sciences, University of Manchester, in compliance with the UK Home Office Animals (Scientific Procedures) Act 1986. C57BL/6 mice were either bred in-house or obtained from Charles River. Age- and sex-matched male or female mice aged 6–20 weeks were used in the experiments.

Mice containing *lox*P sites flanking exon1 of *Mbd2* (*Mbd2*^fl/fl^) were generated using a targeting vector (pBSIIsk+Mbd2_cKO_3_C6) consisting of two overlapping fragments (A and B) covering 9,247 kb of genomic DNA including exon1 of *Mbd2*. Fragment A (2,679 bp) was PCR amplified from genomic DNA from 129 ola mice embryonic stem cells and fragment B (6,683 bp) from BAC DNA (129/Sv mice; Supplementary Fig. 4). Gene targeting with E14TG2a embryonic stem cells was performed as previously described^61^. Correctly targeted clones were identified by PCR screening and confirmed by Southern blotting as performed previously^61^, injected into C57BL/6 blastocysts and transferred to pseudopregnant recipient females. Chimeras were identified by coat colour and tested for germline contribution by mating to C57BL/6 mice. Heterozygotes were bred to produce homozygous *Mbd2*^fl/fl^ mice and genotype was confirmed by Southern blot (Supplementary Fig. 4) and PCR (Supplementary Table 1). Mbd2 protein expression was unaltered in *Mbd2*^fl/fl^ mice, confirmed by western blot using the R593 anti-Mbd2 (ref. 59; Supplementary Fig. 4). To generate animals lacking Mbd2 in CD11c^+^ cells (*Mbd2*^ΔDC^ mice), *Mbd2*^fl/fl^ mice were bred with *Cd11c*-*Cre*^+^ mice^46^, resulting in a mixed 129/Ola × C57BL/6 background. Controls were *Mbd2*^fl/fl^*Cd11c*-*Cre*^*−*^ littermates. *Mbd2* levels in splenic DCs were measured by qPCR (Supplementary Fig. 4).

### Cell culture and isolation

BMDCs were generated with granulocyte–macrophage colony-stimulating factor as previously described^54^ with the omission of 2-ME. In brief, 2 × 10^5^ bone marrow cells were seeded in 10 ml complete medium (RPMI-1640 (Sigma) containing 20 ng ml^−1^ recombinant granulocyte–macrophage colony-stimulating factor (Peprotech), 10% foetal calf serum (Sigma), 2 mM L-glutamine (Gibco), 50 U ml^−1^ penicillin and 50 μg ml^−1^ streptomycin (Life Technologies)). Cells were cultured at 37 °C in a humidified atmosphere of 5% CO_2_. On day 3, 10 ml of complete medium was added and on days 6 and 8, 9 ml of media was gently aspirated and replaced with 10 ml of fresh complete medium. Following 10 days of culture, DCs were harvested and replated at 2 × 10^6^ cells per ml for further assays. To assess morphology, 5 × 10^5^ FACS-sorted CD11c^+^ BMDCs (>95% purity, BD FACS Aria II) were cultured on chambered coverglass (VWR) for 18 h, then fixed, permeabilized and stained with BODIPYFL phallacidin (Life Technologies) and 4,6-diamidino-2-phenylindole in ProLong Fade Gold (Invitrogen) mounting media. Slides were examined by confocal microscopy (Leica SP5 II, 405 and 488 nm, × 63, using LAS AP software), with >50 images assessed for each condition, rendered and analysed using Volocity software (Improvision). To assess antigen uptake, 2 × 10^5^ BMDCs were incubated with 10 μg DQ-OVA (Life Technologies) for 20 min at 37 or 4 °C before staining for flow cytometric analysis. To assess DC responses to antigen exposure, 2 × 10^6^ BMDCs were incubated with 5 μg ml^−1^ heat-killed St (*aro*A attenuated strain of *S. enterica* serovar Typhimurium SL3261) or 25 μg ml^−1^ SEA^54^ for 6 h at 37 °C before flow staining and qPCR as described below.

For some experiments, mononuclear lung and spleen preparations were obtained using a similar method as previously described for splenic single cell suspensions^8^, with incubation at 37 °C for 15 min (spleen) or 30 min (lung). Digested lung was then passed through a 70-μM cell strainer with the aid of a syringe plunger, red blood cells lysed and cells counted before resuspension to the required concentration. Splenic CD11c^hi^MHCII^+^ cDCs were pre-enriched using Dynal DC negative selection kit (Life Technologies) and FACS sorted (>95% purity) for subsequent analysis by qPCR.

### mRNA microarray and reverse transcription qPCR

RNA was extracted from BMDCs using TRIzol, Pure Link RNA Mini Kits and DNase-treated (all from Life Technologies). For microarrays, RNA was then labelled using TotalPrep RNA Amplification kits (Life Technologies) and hybridized with Illumina MouseWG-6 BeadChip arrays (MouseWG6_V2_0_R3_11278593_A) with three biological replicates each for WT and *Mbd2*^*−/−*^ BMDCs. All analyses were conducted in R using Bioconductor^62^. A total of six arrays were QC (quality control) analysed using arrayQualityMetrics in Bioconductor^63^. Raw data that passed QC were transformed using a variance-stabilizing transformation method before normalization across all arrays by the robust spline normalization method, using the lumi package in Bioconductor^64^. Pairwise group comparisons were undertaken using linear modelling. Subsequently, empirical Bayesian analysis was applied, including vertical (within a given comparison) *P* value adjustment for multiple testing, which controls for false-discovery rate, using the limma Bioconductor package. Functional-enrichment analyses were performed for KEGG pathways and GO terms using the appropriate packages. Focused ‘genes of interest’ lists were assembled from the literature and other publically available resources. The microarray data discussed in this publication were deposited in NCBI’s Gene Expression Omnibus^65^, accessible through GEO Series accession number GSE66096.

For RT–qPCR of tissue, complementary DNA was generated from extracted RNA using SuperScript-III and Oligo-dT (all from Life Technologies). Relative quantification of genes of interest was performed by qPCR analysis using Roche LightCycler 480, with LightCycler SYBR Green I Master mix, compared with a serially diluted standard of pooled complementary DNA. Expression was normalized to hypoxanthine–guanine phosphoribosyltransferase (*Hprt*) or (*Gapdh*). Primers are listed in Supplementary Table 1.

### Chromatin immunoprecipitation and sequencing

ChIP was performed as described previously^66^ with modifications. Cross-linked chromatin from 3–6 × 10^6^ BMDCs was incubated with anti-IgG (Abcam) or anti-H3ac (Millipore). Immunoprecipitated DNA was analysed by high throughput Illumina HiSeq 2,000 sequencing, following standard protocols. Illumina data were mapped to the mouse genome (NCBIm37) using BWA^67^ with reads mapping to multiple locations filtered out. Mapped sequence data (WIG files) were analysed using tools developed for DNA methylation analysis^68^, based on R and perl scripts interfaced with the Galaxy server. Raw sequencing data was normalized to the average number of mapped bases in each sample to account for variable sequence depth between samples. ChIP sequencing density was determined by calculating the average number of hits per base in 100-bp windows with a 20-bp slide using normalized.WIG files over a 6-kb interval centred on the TSS (for promoters). Composite profiles were generated by plotting median values in each window for each sample.

### *In vitro* and *in vivo* T-cell activation and polarization

To assess T-cell proliferation, CD4^+^ T cells were negatively selected from the spleen and LN of OT-IIxLy5.1 T-cell receptor transgenic mice (Dynal, Life Technologies), labelled with CFSE (carboxyfluorescein succinimidyl ester) as described previously^16^, and cultured with 5 × 10^4^ WT or *Mbd2*^−/−^ BMDCs in the presence of 0.01 μg ml^−1^ OVA_323–339_ peptide or 5 μg ml^−1^ OVA protein (Sigma; endotoxin depleted in-house). Cultures were incubated at 37 °C for 4 days before assessment of CFSE dilution by flow cytometry.

For measurement of DC–T-cell adhesion, based on previous work^69^, WT or *Mbd2*^*−/−*^ BMDCs were seeded onto Ibidi μ-slide VI^0.4^ plates (30 μl per well at 6 × 10^6^ cells) and adhered for 18 h. CD4^+^ T cells were positively selected from WT spleens and LN (Miltenyi Biotec). T cells (1 × 10^6^ cells per ml in adhesion medium; RPMI plus 0.1% BSA, 40 mM HEPES and 2 mM MgCl_2_) were stimulated with 200 nM PdBu for 5 min to activate their integrins, and flowed over the DCs at a rate of 0.3 dynes per cm^2^ using a Multi-phaser NE-1,000 (New Era Pump Systems Inc.) for 10 min, with manual counting of adhered T cells in the field of view at 2-min intervals.

For DC polarization of T cells *in vitro*, CD4^+^GFP^*−*^ T cells were FACS sorted from KN2xIL-10eGFP, KN2xIL-13eGFP or IL-10eGFP mice and cultured with WT or *Mbd2*^−/−^ BMDCs, 1 μg ml^−1^ anti-CD3 (grown in-house), in the presence or absence of IL-4 (20 ng ml^−1^, Peprotech) for Th2 conditions, as described previously^16^, or IL-12 (20 ng ml^−1^, Peprotech) for Th1 conditions, or IL-6 (20 ng ml^−1^), transforming growth factor-β (1 ng ml^−1^) and IL-23 (10 ng ml^−1^, all Peprotech) for Th17 conditions. To assess Th1/Th17 or Th2 priming *in vivo*, 25 μg heat-killed St or 2.5 × 10^4^ *S. mansoni* eggs were injected subcutaneously per foot into recipient mice. Draining popliteal LNs were harvested 7 days later, and restimulated as described previously^54^,^70^. In some experiments, mice were injected subcutaneously with 2.5 × 10^5^ BMDCs that had been cultured for 6 h with 25 μg ml^−1^ SEA or medium alone. popliteal LNs were harvested 7 days later and cultured as described above. In other experiments, based on previous work^51^, BMDCs were cultured for 18 h with 100 μg ml^−1^ HDM (*Dermatophagoides farina, Df*, Greer Laboratories). Mice were then sensitized intranasally with 1 × 10^4^ BMDCs in 50 μl PBS, challenged with 5 μg *Df* intranasally on days 14 and 15 and euthanized on day 17. Cytospins were prepared from BAL for differential cell counts (200 cells per slide), following Diff Quick staining (Reagena). Standard hematoxylin and eosin stain staining was performed on the lung lobe sections to assess gross pathology, and portions of the lung collected into TRIzol for RNA extraction and qPCR.

### Flow cytometry and ELISA

Cells were first stained with LiveDead aqua or blue (Life Technologies). Following FcR-Block (2.4G2), cells were stained using the following monoclonal antibodies (all used at 1:200 dilution): CD3-APCe780, CD4-APC or A700, CD8α-PE/Cy7, CD11c-APC or BV421, MHC-II-PerCP/Cy5.5 or eFlour450, CD11b-BV711 or PE, CD19-e780, CD80-PerCP/Cy5.5, CD40-PE, CD44-BV570, CD69-FITC CD86-AF488, Foxp3-e450, F4/80-PE/Cy7, Gr1-FITC, HuCD2-PE, IgM-APCe780, SiglecF-PE and TCRβ-APCe780 (BD Biosciences, BioLegend or eBioscience). Foxp3 staining was performed with the eBioscience FoxP3 staining kit. Samples were acquired using FACS LSR II or FACS Canto II using BD FACSDiva software and analysed with FlowJo v.9 software (Tree Star). Cytokines were measured in culture supernatants by ELISA using paired monoclonal antibody, and recombinant cytokine standards, or Duosets (eBioscience, BD Biosciences, BioLegend, R&D Systems and Peprotech).

### Statistical analysis

Statistical analyses were carried out using GraphPad Prism 6. The Student’s *t*-test or ANOVA (analysis of variance) was used to determine significant differences between the sample groups (in figures, *P<0.05, **P<0.01, ***P<0.001 and ****P<0.0001).

## Additional information

**How to cite this article:** Cook, P. C. *et al*. A dominant role for the methyl-CpG-binding protein Mbd2 in controlling Th2 induction by dendritic cells. *Nat. Commun.* 6:6920 doi: 10.1038/ncomms7920 (2015).

## Supplementary Material

Supplementary Figures and Tables Supplementary Figures 1-5 and Supplementary Table 1

Supplementary Data 1 Microarray analysis of gene expression, comparing Mbd2-/- DCs to WT

Supplementary Data 2 GO term enrichment analysis of mRNA expression data from Mbd2-/- versus WT DCs (related to Fig. 1).

Supplementary Data 3 List of annotated TSS from ChIP-seq heat map profile of H4K9/K14ac signal, comparing significantly down-regulated mRNA profiles of Mbd2-/- DCs to WT DCs (related to Supplementary Figure 2).

Supplementary Data 4 List of annotated TSS from ChIP-seq heat map profile of H4K9/K14ac signal, comparing significantly up-regulated mRNA profiles of Mbd2-/- DCs to WT DCs (related to Supplementary Figure 2).
