# Supplementary material for: A dominant role for the methyl-CpG-binding protein Mbd2 in controlling Th2 induction by dendritic cells
Source: Nat Commun. 2015 Apr 24;6:6920. doi: 10.1038/ncomms7920 (PMC4413429; doi:10.1038/ncomms7920)
Supplement: Supplementary Figures and Tables — Supplementary Figures 1-5 and Supplementary Table 1 [file ncomms7920-s1.pdf]

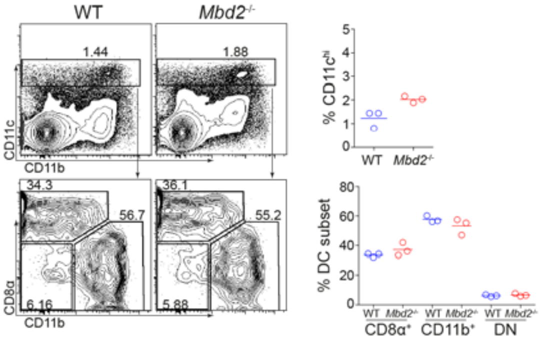

Supplementary Figure 1. **Mbd2 is not required for DC development *in vivo*.**

The proportion of splenic DC subsets from WT or *Mbd2*$^{-/-}$ mice was assessed by flow cytometry. Dead cells, doublets, and CD19+ NK1.1+ Gr1+ cells were excluded (3 mice per group, one of two experiments).

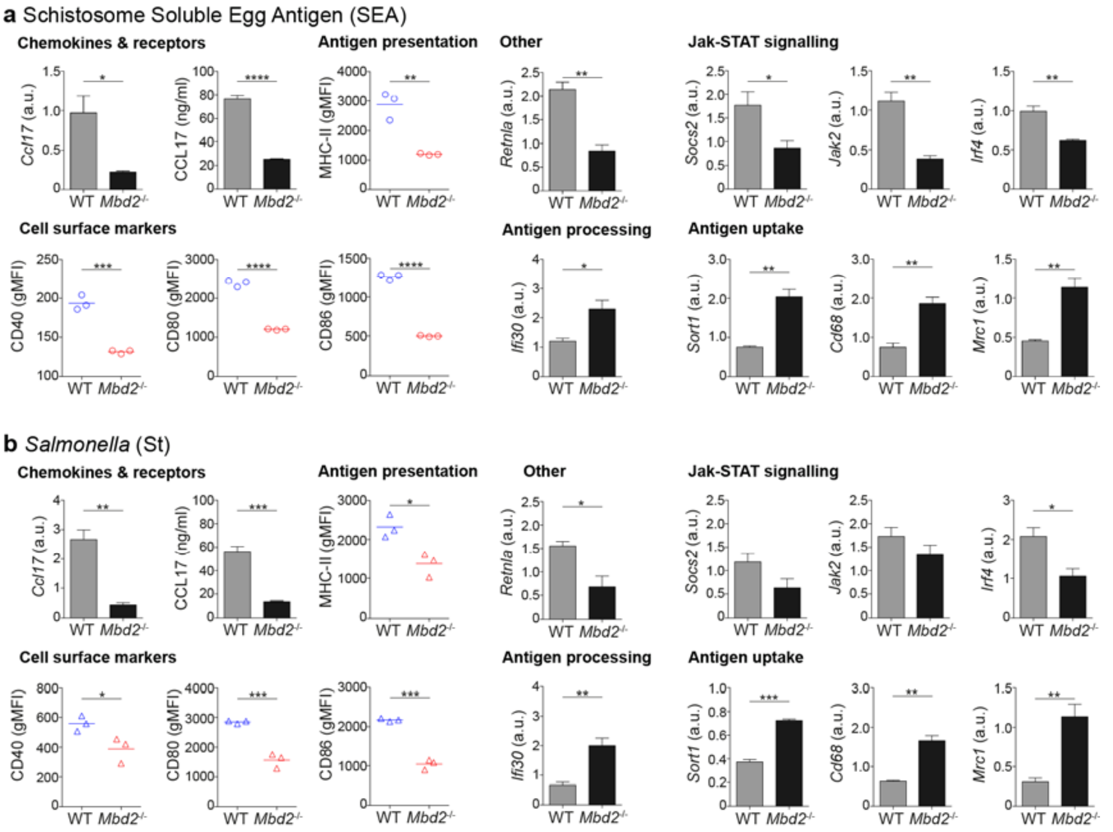

Supplementary Figure 2. Mbd2 regulates expression of key pathways following DC stimulation with helminth or bacterial antigens (related to Figure 1).

WT (grey) or *Mbd2*$^{-/-}$ (black) BMDCs were incubated with (a) SEA or (b) heat-killed St for 6 h to measure mRNA expression of genes of interest by qPCR (normalized against *Hprt*, a.u.), or overnight to measure surface protein expression by flow cytometry. Results are mean + SEM (3 replicate wells, one of at least three experiments). **P*<0.05, ***P*<0.01, ****P*<0.001, *****P*<0.0001 (Student's t test). a.u. = arbitrary units, gMFI = geometric mean fluorescence intensity.

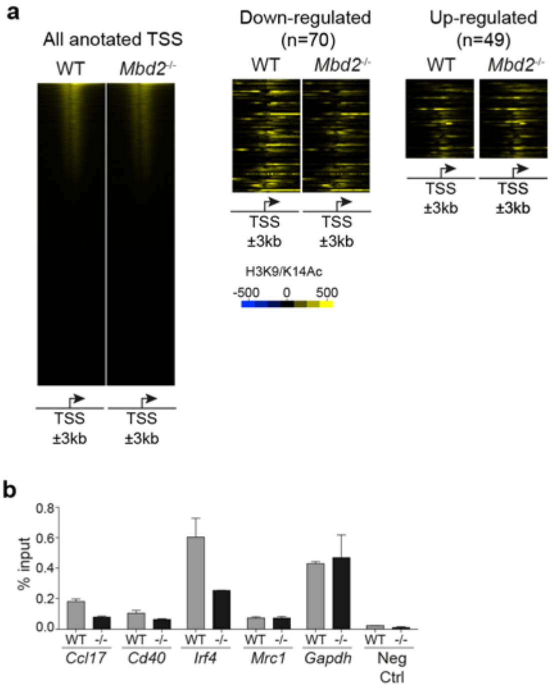

Supplementary Figure 3. ***Mbd2*$^{-/-}$ DCs display altered H3 acetylation of dysregulated genes (related to Fig. 2).**

(a) ChIP-seq heat map profile showing H3K9/K14ac signal for WT and *Mbd2*$^{-/-}$ BMDCs for all annotated TSS and for genes with significantly altered mRNA profiles as shown in Fig. 1. Signal is displayed from -3 kb to +3 kb surrounding each annotated TSS.

(b) ChIP-qPCR using anti-H3K9/K14ac antibody to validate ChIP-seq data in Fig. 2. Immunoprecipitated and input DNA was analysed by qPCR using primers to genes Ccl17, Cd40, Irf4, Mrc1, Gapdh (positive control) and m15 (negative control, corresponding to an intergenic region on chromosome 15). 3 technical replicates, from one of 7 ChIP-qPCR analyses of 3 separate BMDC experiments.

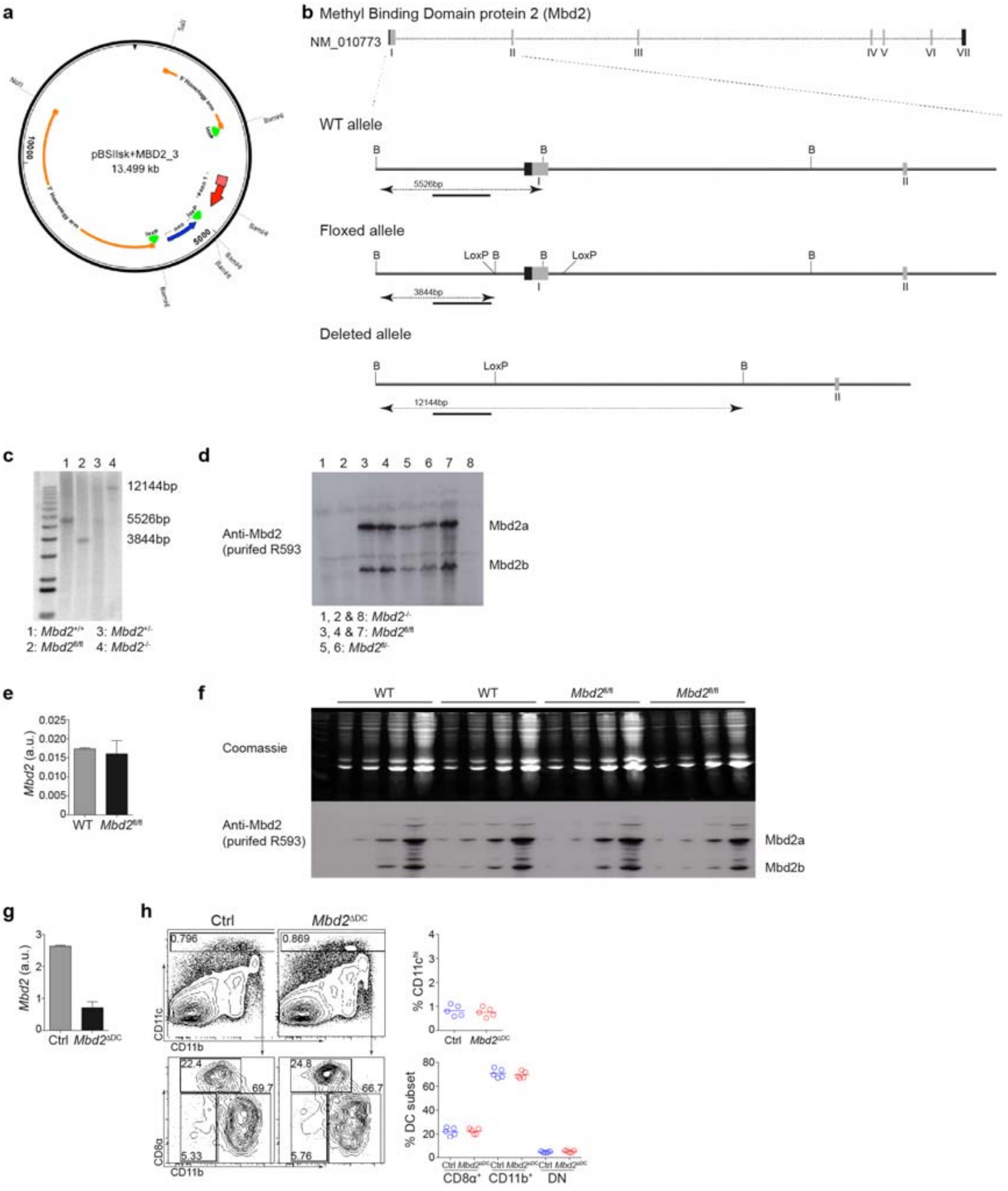

Supplementary Figure 4. CD11c-specific deletion of Mbd2 does not alter DC development.

(a) Schematic of the targeting vector used to create the *Mbd2*$^{fl/fl}$ mice.

(b) Schematic of the targeting vector and Mbd2 locus: 7 exons are indicated, black bars are 5' and 3' UTR and grey bars are coding sequences. Detailed view of WT allele, exon 1 (I), exon 2 (II) and BamH1 (B) sites are shown. The position of the southern blot probe (black bar) and the size of BamH1 fragment that binds to the probe (dotted arrow) are shown. The probe is a 2059 bp Sal1/BamH1 fragment of Mbd2 of the targeting vector pBSIIsk+MBD2cKO_3_c6. The Floxed allele was created by insertion of two loxP sites 5' and 3' of exon 1. Intercross between *Mbd2*$^{fl/fl}$ strain and CD11c-Cre^{+} knockin strain facilitated the generation of CD11c-specific *Mbd2*$^{-/-}$ mice (*Mbd2*$^{\Delta DC}$).

(c) Southern blot analysis using a radio labelled Sal1/BamH1 fragment from the targeting vector showing the expected sizes of WT (*Mbd2*$^{+/+}$,lane 1), *Mbd2*$^{fl/fl}$ (lane 2), *Mbd2*$^{fl/-}$ (lane 3) and *Mbd2*$^{-/-}$ (lane 4) bands using genomic DNA isolated from kidney tissue or tail clips.
(d) Western blot analysis using anti-Mbd2 R593 antibodies showing Mbd2 protein expression from splenic extracts isolated from *Mbd2*$^{-/-}$ (lane 1, 2 and 8), *Mbd2*$^{fl/fl}$ (lane 3, 4 and 7) and *Mbd2*$^{fl/-}$ (lane 5 and 6) mice. Each lane represents an individual mouse.
(e) Transcript expression of Mbd2 from spleens of WT and Mbd2$^{fl/fl}$ mice was assessed by qPCR (normalized against *Gapdh*, a.u.). Results are mean + SEM (2 mice per group).
(f) Western blot analysis using anti-Mbd2 R593 antibodies (lower panel) showing Mbd2 protein expression from splenic extracts isolated from WT and *Mbd2*$^{fl/fl}$ mice (same mice as in e). Upper panel is the coomassie stained gel of total protein. A series dilution was used for each protein extract.
(g) mRNA expression of Mbd2 in FACS- sorted splenic CD11c^{hi}MHCII^{+} DCs from *Mbd2*$^{\Delta DC}$ or littermate control mice was assessed by qPCR (normalised against Gapdh, a.u.; 3 replicates from pooled spleens of 5 mice, from one of two experiments).
(h) The proportion of splenic DC subsets from *Mbd2*$^{\Delta DC}$ or littermate controls mice assessed by flow cytometry. Dead cells, doublets, CD19+ NK1.1+ Gr1+ cells were excluded (3 mice per group, representative of at least three experiments).

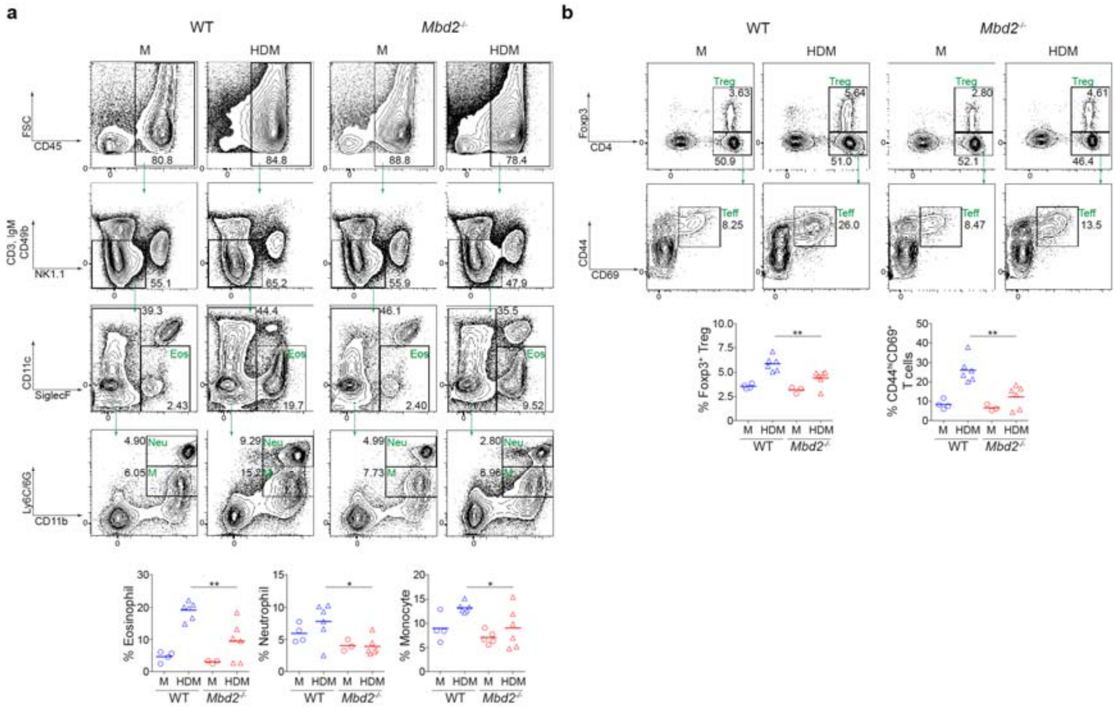

Supplementary Figure 5. Flow cytometry gating scheme to identify innate cells and T cells from lung tissue (related to Fig. 6).

WT mice were sensitized by intranasal administration of WT (blue) or *Mbd2*$^{-/-}$ (red) BMDCs cultured overnight in medium alone (M) or with HDM, then challenged intranasally with 5 µg HDM on d 14 and d 15 post DC transfer. Tissues were harvested on d 17.

(a) Proportions of eosinophils (Eos), neutrophils (Neu) and monocytes (M) in lung tissue were assessed by flow cytometry. Dead cells and doublets excluded, all percentages shown relate to proportion of live cells (3-6 mice per group, one of six experiments).

(b) Proportions of TCRβ$^+$CD4$^+$Foxp3$^+$ Treg cells (Treg) and activated effector TCRβ$^+$CD4$^+$Foxp3$^-$CD44$^+$CD69$^+$ T cells (Teff) in lung tissues were assessed by flow cytometry. Gating strategy for these populations is shown. Dead cells, doublets and TCRβ$^-$ cells excluded (3-6 mice per group, one of six experiments).

*P<0.05, **P<0.01 (ANOVA).

Supplementary Table 1. Primer list.

Mbd2$^{fl/fl}$ genotyping primers
3F: TCTTCCCAAGTTGCTTTTG
3R: TGAGGTCTCTCGGATGGAAC
3F/3R: 178bp band (WT) / 223bp (Mbd2^{fl} allele)
5R: GGCGTTATAACTTCGTATAGC
3F/5R: 178bp band (WT), 223bp (Mbd2^{fl} allele)

RT-qPCR primers
Il-4[21]
Il-13 F: CCTCTGACCCTTAAGGAGCTTAT
Il-13 R: CGTTGCACAGGGGAGTCT
Ccl17 F: TGCTTCTGGGGACTTTTCTG
Ccl17 R: GAATGGCCCCTTTGAAGTAA
Irf4 F: ACAGCACCTTATGGCTCTCTG
Irf4 R: ATGGGGTGGCATCATGTAGT
Mbd2 F: CCTTAGCAGTTTTGACTTCAGG
Mbd2 R: TGGCAATGTTGTGTTCAGGT
Jak2 F: AAGATTGCCAAGGCCAGA
Jak2 R: TGTTGTTCCAGCACTCTGTCA
Retnla[21]
Socs2 F: CGCGAGCTCAGTCAAACAG
Socs2 R: AGTTCCTTCTGGAGCCTCTTTT
Mrc1[21]
Hprt[21]
Gapdh[11]
Ifi30 F: GCGTTCCTAACCATCGTCTG
Ifi30 R: GTGACACCTCAGGAGCATACAC
Sort1 F: TGAGGACATGGTCTTCATGC
Sort1 R: GGTAAAGATGGTGCCAAACC
CD68 F: GACCTACATCAGAGCCCGAGT
CD68 R: CGCCATGAATGTCCACTG
